# Supplementary material for: An Improved Synthetic Method for Sensitive Iodine Containing Tricyclic Flavonoids
Source: Molecules. 2022 Dec 2;27(23):8430. doi: 10.3390/molecules27238430 (PMC9740535; doi:10.3390/molecules27238430)
Supplement: Supplementary file 1 [file molecules-27-08430-s001.zip › molecules-2060930-supplementary.pdf]

An Improved Synthetic Method for Sensitive Iodine Containing Tricyclic Flavonoids

Mihail Lucian Birsa and Laura G. Sarbu

Supplementary Material

1. Elemental analysis **S2**

2. Nuclear magnetic resonance data **S3-S4**

2. Copies of ^{13}C NMR spectra **S5-S14**

1. Elemental analysis

Elemental analyses (C, H) were conducted using a CE440 Elemental Analyser; the results were found to be in good agreement (±0.3%) with the calculated values.

Table S1. Elemental analysis data for compounds **2**, **4a**–**e** and **5a**–**e**.

Compound	% C		% H	
	calcd.	found	calcd.	found
**2**	29.17	29.31	2.82	3.08
**4a**	39.57	39.74	3.32	3.47
**4b**	40.57	40.81	3.56	3.77
**4c**	37.46	37.65	2.83	3.02
**4d**	34.21	34.45	2.58	2.74
**4e**	38.61	38.85	3.24	3.41
**5a**	35.67	35.87	2.85	3.07
**5b**	36.64	36.88	3.07	3.31
**5c**	33.78	34.02	2.41	2.64
**5d**	31.12	31.40	2.22	2.47
**5e**	34.88	35.15	2.79	3.01

Table S2. Nuclear magnetic resonance data for compounds **2**, **4a**–**e** and **5a**–**e**.

Compound	^{1}H NMR and ^{13}C NMR data
**2**	^{1}H NMR ($CDCl_3$) δ 12.71 (s, 1H), 8.30 (d, *J* = 1.6 Hz, 1H), 8.25 (d, *J* = 1.7 Hz, 1H), 4.86 (s, 2H), 4.03 (q, *J* = 6.9 Hz, 2H), 3.83 (q, *J* = 6.9 Hz, 2H), 1.38 (t, *J* = 6.9 Hz, 3H), 1.30 (t, *J* = 6.9 Hz, 3H).<br>^{13}C NMR ($CDCl_3$) δ 198.1, 192.8, 160.6, 152.9, 138.7, 120.6, 88.2, 80.6, 50.5, 47.3, 43.5, 12.6, 11.5.
**4a**	^{1}H NMR ($CDCl_3$, selected data for the major isomer) δ 8.26 (d, *J* = 1.7 Hz, 1H), 8.13 (d, *J* = 1.7 Hz, 1H), 7.38 (d, *J* = 7.7 Hz, 2H), 7.16 (d, *J* = 7.7 Hz, 2H), 6.01 (d, *J* = 6.2 Hz, 1H), 5.75 (d, *J* = 6.2 Hz, 1H), 3.99 (m, 2H), 3.68 (m, 2H), 2.35 (s, 3H), 1.25 (t, *J* = 6.9 Hz, 6H).<br>^{13}C NMR ($CDCl_3$, selected data for the major isomer) δ 191.6, 186.2, 158.8, 152.6, 138.7, 136.3, 132.8, 129.3, 127.2, 122.9, 87.6, 84.7, 83.0, 57.8, 50.5, 47.3, 21.2, 12.6, 11.4.
**4b**	^{1}H NMR ($CDCl_3$, selected data for the major isomer) δ 8.26 (d, *J* = 1.8 Hz, 1H), 8.13 (d, *J* = 1.8 Hz, 1H), 7.4 (d, *J* = 7.9 Hz, 2H), 7.19 (d, *J* = 7.9 Hz, 2H), 6.01 (d, *J* = 6.3 Hz, 1H), 5.76 (d, *J* = 6.3 Hz, 1H), 3.98 (m, 2H), 3.69 (m, 2H), 2.65 (q, *J* = 7.5 Hz, 2H), 1.25 (t, *J* = 7.5 Hz, 3H), 1.23 (t, *J* = 7.6 Hz, 6H).<br>^{13}C NMR ($CDCl_3$, selected data for the major isomer) δ 191.2, 186.3, 158.8, 152.6, 144.9, 136.3, 133.0, 128.1, 127.2, 122.9, 87.6, 84.7, 83.0, 57.8, 50.5, 47.3, 28.5, 15.3, 12.6, 11.4.
**4c**	^{1}H NMR ($CDCl_3$, selected data for the major isomer) δ 8.27 (d, *J* = 1.9 Hz, 1H), 8.16 (d, *J* = 1.9 Hz, 1H), 7.49 (m, 2H), 7.06 (m, 2H), 5.96 (d, *J* = 7.3 Hz, 1H), 5.79 (d, *J* = 7.3 Hz, 1H), 3.95 (m, 2H), 3.71 (m, 2H), 1.24 (t, *J* = 6.8 Hz, 6H).<br>^{13}C NMR ($CDCl_3$, selected data for the major isomer) δ 190.9, 186.1, 162.9, 158.7, 152.7, 136.4, 131.7, 129.4, 122.4, 115.5, 87.4, 84.9, 82.6, 58.4, 50.7, 47.3, 12.6, 11.4.
**4d**	^{1}H NMR ($CDCl_3$, selected data for the major isomer) δ 8.28 (d, *J* = 1.9 Hz, 1H), 8.16 (d, *J* = 1.9 Hz, 1H), 7.52 (d, *J* = 8.2 Hz, 2H), 7.41 (d, *J* = 8.2 Hz, 2H), 5.95 (d, *J* = 7.1 Hz, 1H), 5.74 (d, *J* = 7.1, 1H), 3.92 (m, 2H), 3.66 (m, 2H), 1.24 (t, *J* = 6.7 Hz, 6H).<br>^{13}C NMR ($CDCl_3$, selected data for the major isomer) δ 190.8, 185.9, 158.7, 152.7, 136.5, 134.8, 131.7, 129.1, 123.1, 122.1, 87.4, 85.1, 82.6, 58.2, 50.7, 47.4, 12.6, 11.4.

**4e**	^{1}H NMR ($CDCl_3$, selected data for the major isomer) δ 8.26 (m, 1H), 8.14 (m, 1H), 7.42 (d, *J* = 8.2 Hz, 2H), 6.89 (d, *J* = 8.2 Hz, 2H), 5.96 (d, *J* = 6.7 Hz, 1H), 5.78 (d, *J* = 6.7 Hz, 1H), 3.97 (m, 2H), 3.81 (s, 3H), 3.65 (m, 2H), 1.25 (t, *J* = 6.7 Hz, 6H).<br><br>^{13}C NMR ($CDCl_3$, selected data for the major isomer) δ 191.2, 186.4, 159.9, 158.8, 152.6, 136.4, 128.7, 127.9, 122.8, 113.9, 87.7, 87.6, 84.7, 58.1, 55.3, 50.5, 47.3, 12.6, 11.4.
**5a**	^{1}H NMR (DMSO-*d*6) δ 8.04 (d, *J* = 1.8 Hz, 1H), 7.74 (d, *J* = 1.8 Hz, 1H), 7.37 (d, *J* = 8.0 Hz, 2H), 7.26 (d, *J* = 8.0 Hz, 2H), 6.89 (s, 1H), 3.89 (m, 4H), 2.31 (s, 3H), 1.40 (t, *J* = 7.1 Hz, 3H), 1.32 (t, *J* = 7.1 Hz, 3H).<br><br>^{13}C NMR (DMSO-*d*6) δ 184.9, 150.7, 147.8, 140.2, 133.8, 130.0, 129.7, 127.8, 126.8, 119.1, 88.7, 87.6, 76.2, 54.7, 54.6, 21.3, 10.8, 10.5.
**5b**	^{1}H NMR (DMSO-*d*6) δ 8.06 (d, *J* = 1.8 Hz, 1H), 7.73 (d, *J* = 1.8 Hz, 1H), 7.35 (d, *J* = 8.1 Hz, 2H), 7.24 (d, *J* = 8.1 Hz, 2H), 6.87 (s, 1H), 3.87 (m, 4H), 2.35 (q, *J* = 7.3 Hz, 2H), 1.40 (t, *J* = 7.1 Hz, 3H), 1.32 (t, *J* = 7.1 Hz, 3H), 1.26 (t, *J* = 7.3 Hz, 3H).<br><br>^{13}C NMR (DMSO-*d*6) δ 185, 150.5, 147.7, 140.1, 133.5, 129.9, 129.6, 127.5, 126.7, 119.0, 88.8, 87.5, 76.1, 54.8, 54.6, 25.4, 12.3, 10.7, 10.4.
**5c**	^{1}H NMR (DMSO-*d*6) δ 8.09 (d, *J* = 1.8 Hz, 1H), 7.80 (d, *J* = 1.8 Hz, 1H), 7.55 (dd, $^{3}J_{H\text{-}H}$ = 8.7 Hz, $^{4}J_{H\text{-}F}$ = 5.3 Hz, 2H), 7.30 (dd, $^{3}J_{H\text{-}H}$ = 8.8 Hz, $^{3}J_{H\text{-}F}$ = 8.7 Hz, 2H), 6.96 (s, 1H), 3.90 (m, 4H), 1.40 (t, *J* = 7.1 Hz, 3H), 1.33 (t, *J* = 7.1 Hz, 3H).<br><br>^{13}C NMR (DMSO-*d*6) δ 185.0, 164.2, 162.3, 150.4, 147.8, 133.0, 130.3, 129.2, 127.1, 119.0, 116.5, 88.7, 87.7, 75.5, 54.7, 54.6, 10.8, 10.5.
**5d**	^{1}H NMR (DMSO-*d*6) δ 8.09 (d, *J* = 1.7 Hz, 1H), 7.79 (d, *J* = 1.7 Hz, 1H), 7.66 (d, *J* = 8.4 Hz, 2H), 7.43 (d, *J* = 8.4 Hz, 2H), 6.92 (s, 1H), 3.87 (m, 4H), 1.39 (t, *J* = 7.1 Hz, 3H), 1.32 (t, *J* = 7.1 Hz, 3H).<br><br>^{13}C NMR (DMSO-*d*6) δ 185.0, 150.4, 147.9, 136.0, 133.1, 132.5, 130.0, 128.7, 127.2, 123.9, 119.0, 88.6, 87.8, 75.5, 54.7, 54.6, 10.7, 10.5.
**5e**	^{1}H NMR (DMSO-*d*6) δ 8.08 (d, *J* = 1.8 Hz, 1H), 7.78 (d, *J* = 1.8 Hz, 1H), 7.41 (d, *J* = 8.7 Hz, 2H), 6.99 (d, *J* = 8.7 Hz, 2H), 6.86 (s, 1H), 3.90 (m, 4H), 3.76 (s, 3H), 1.40 (t, *J* = 7.1 Hz, 3H), 1.32 (t, *J* = 7.1 Hz, 3H).<br><br>^{13}C NMR (DMSO-*d*6) δ 184.9, 160.9, 150.7, 147.7, 132.9, 129.9, 129.6, 128.6, 126.7, 119.0, 114.8, 88.7, 87.4, 76.1, 55.7, 54.7, 54.6, 10.8, 10.5.

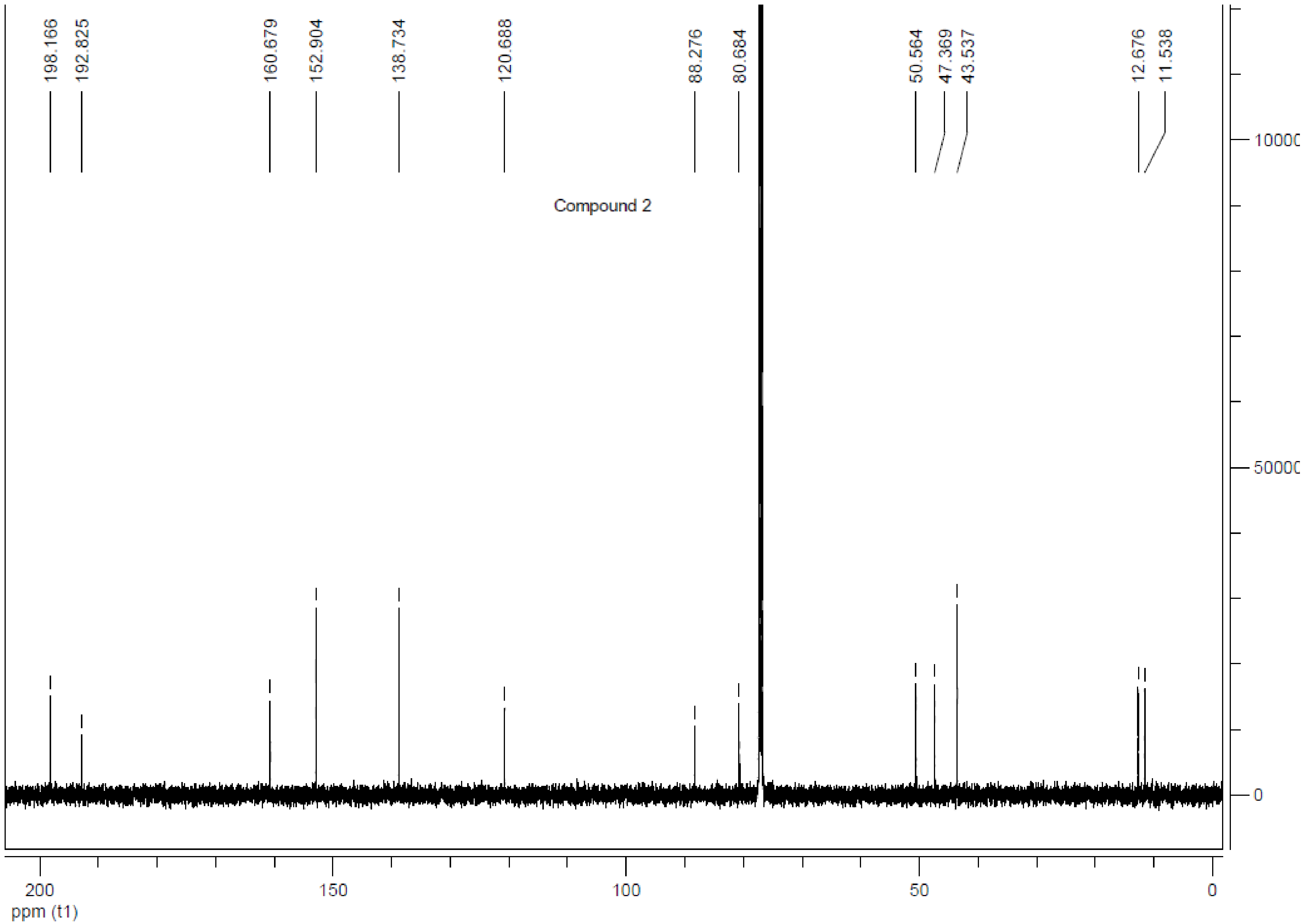
198.166
192.825
160.679
152.904
138.734
120.688
88.276
80.684
50.564
47.369
43.537
12.676
11.538
Compound 2
10000
50000
0
200
150
100
50
0
ppm (t1)

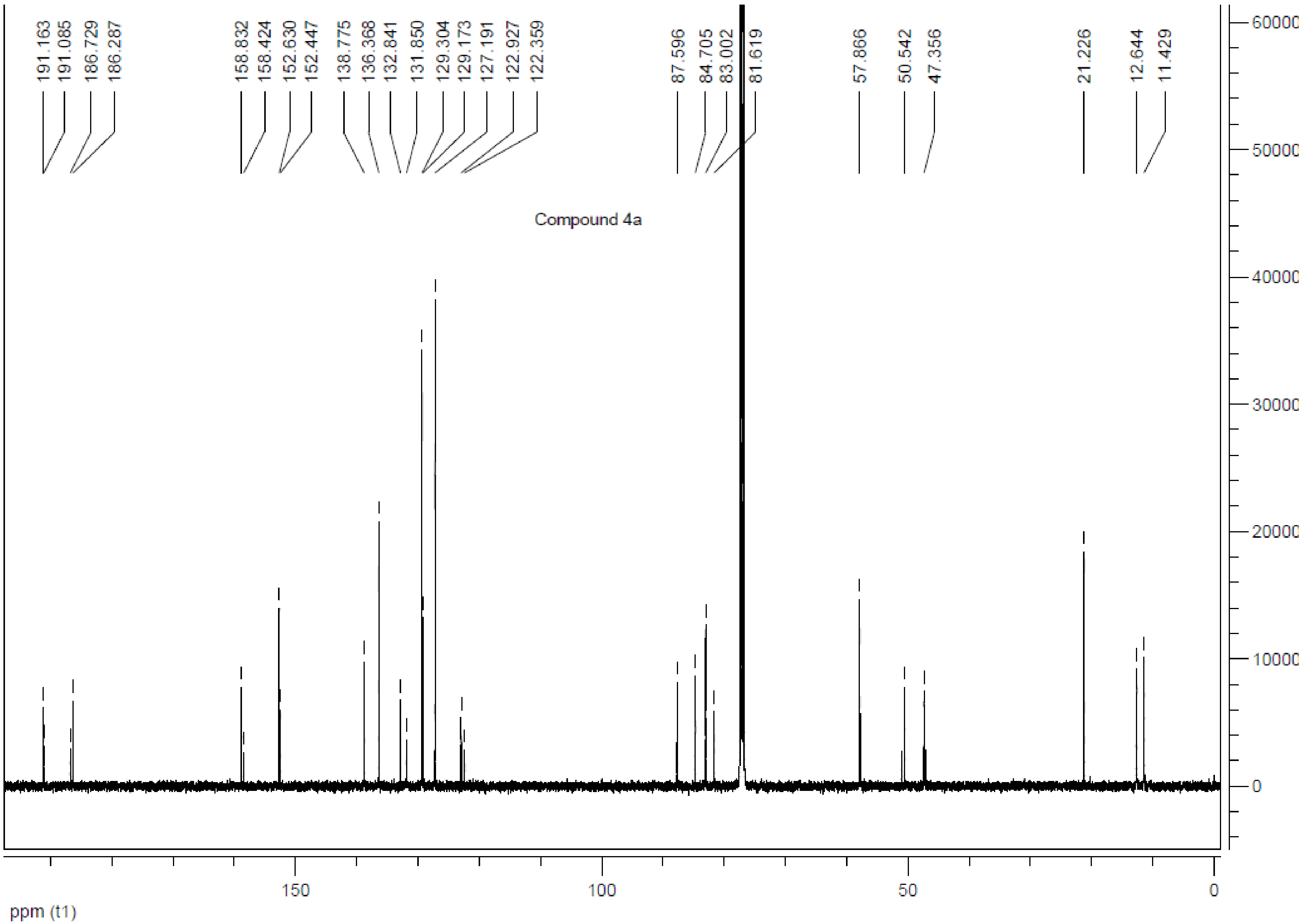

191.163
191.085
186.729
186.287
158.832
158.424
152.630
152.447
138.775
136.368
132.841
131.850
129.304
129.173
127.191
122.927
122.359
87.596
84.705
83.002
81.619
57.866
50.542
47.356
21.226
12.644
11.429
Compound 4a
60000
50000
40000
30000
20000
10000
0
150
100
50
0
ppm (t1)

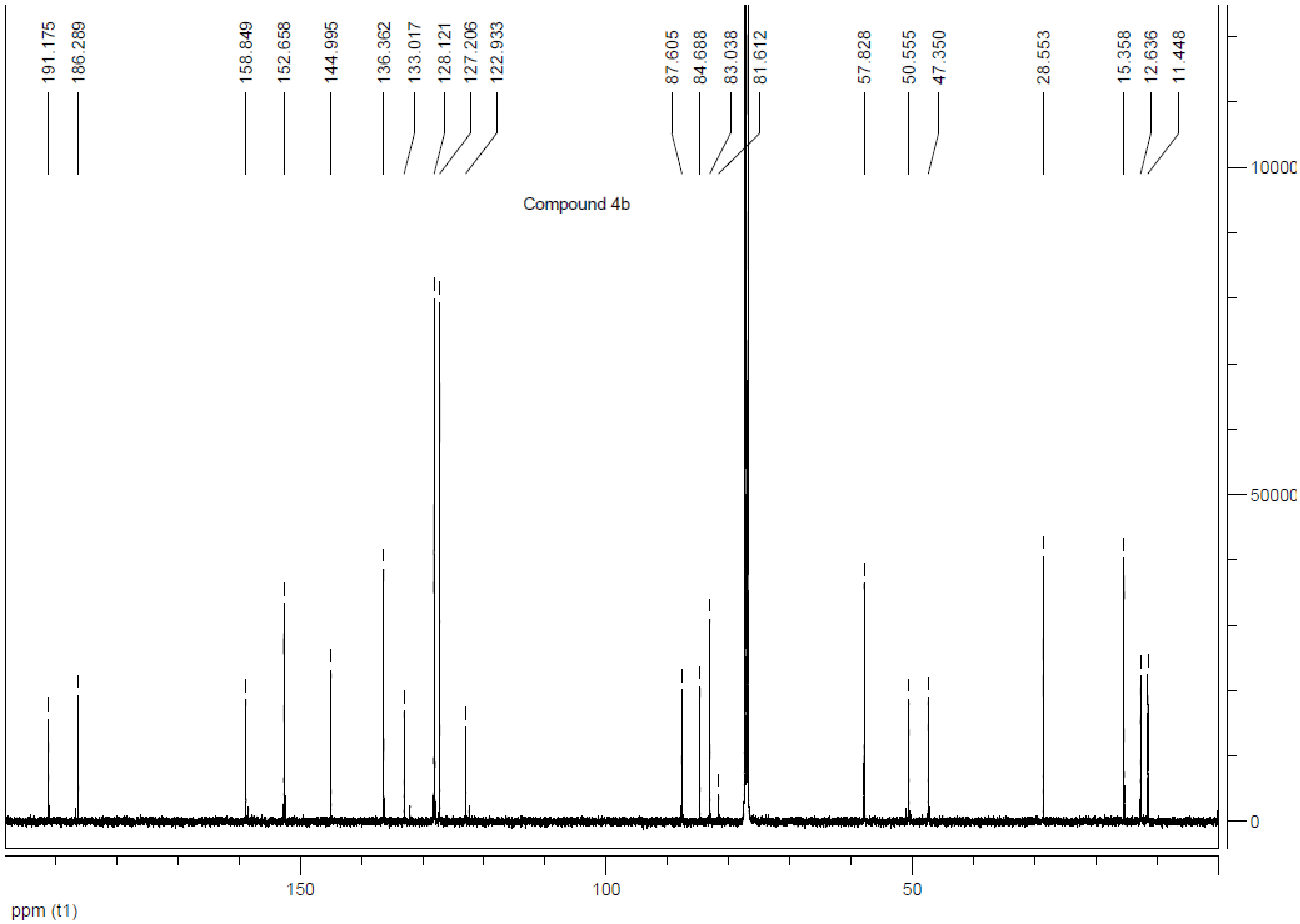

191.175
186.289
158.849
152.658
144.995
136.362
133.017
128.121
127.206
122.933
87.605
84.688
83.038
81.612
57.828
50.555
47.350
28.553
15.358
12.636
11.448
Compound 4b
10000
50000
0
150
100
50
ppm (t1)

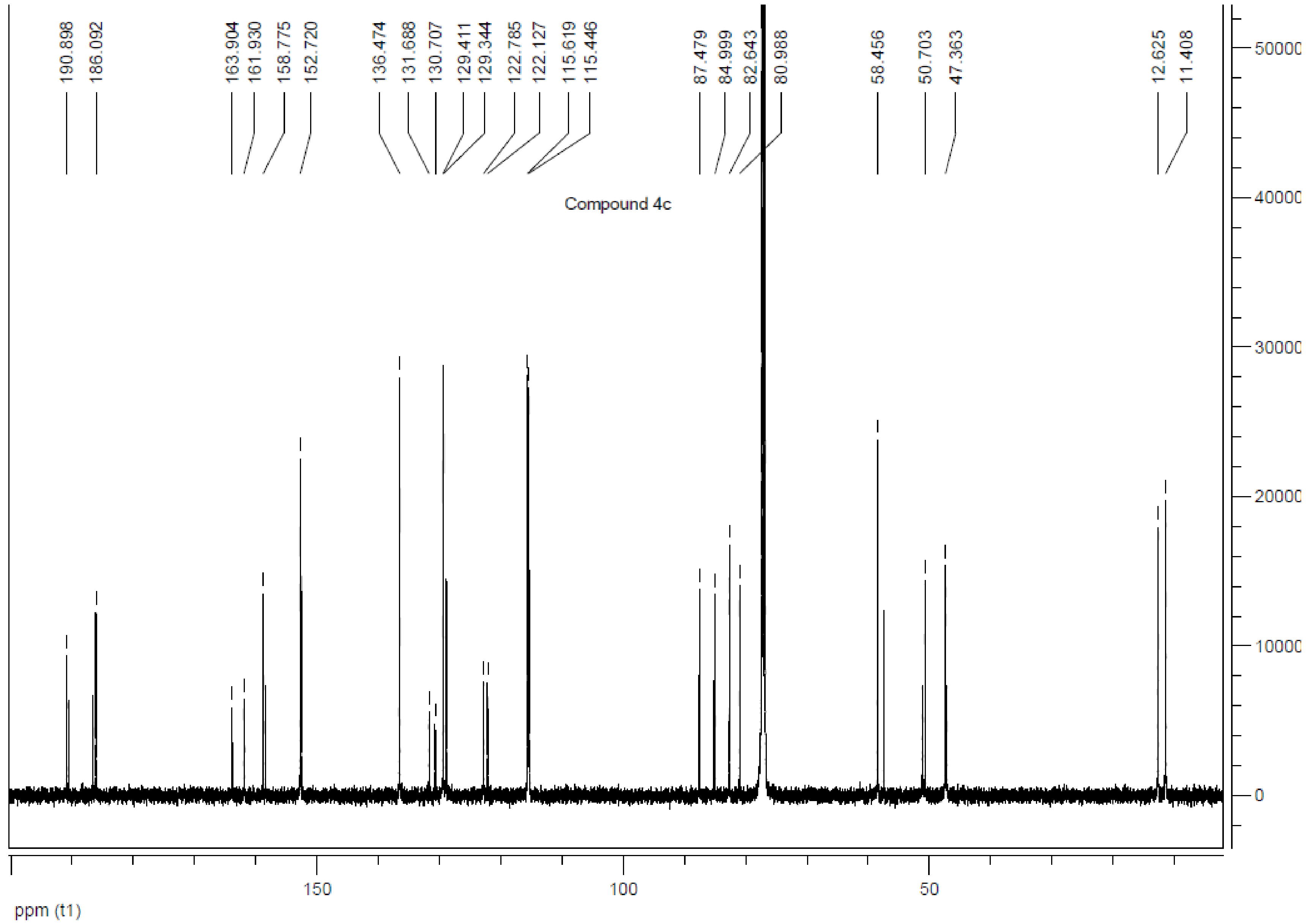
190.898
186.092
163.904
161.930
158.775
152.720
136.474
131.688
130.707
129.411
129.344
122.785
122.127
115.619
115.446
87.479
84.999
82.643
80.988
58.456
50.703
47.363
12.625
11.408
Compound 4c
50000
40000
30000
20000
10000
0
150
100
50
ppm (t1)

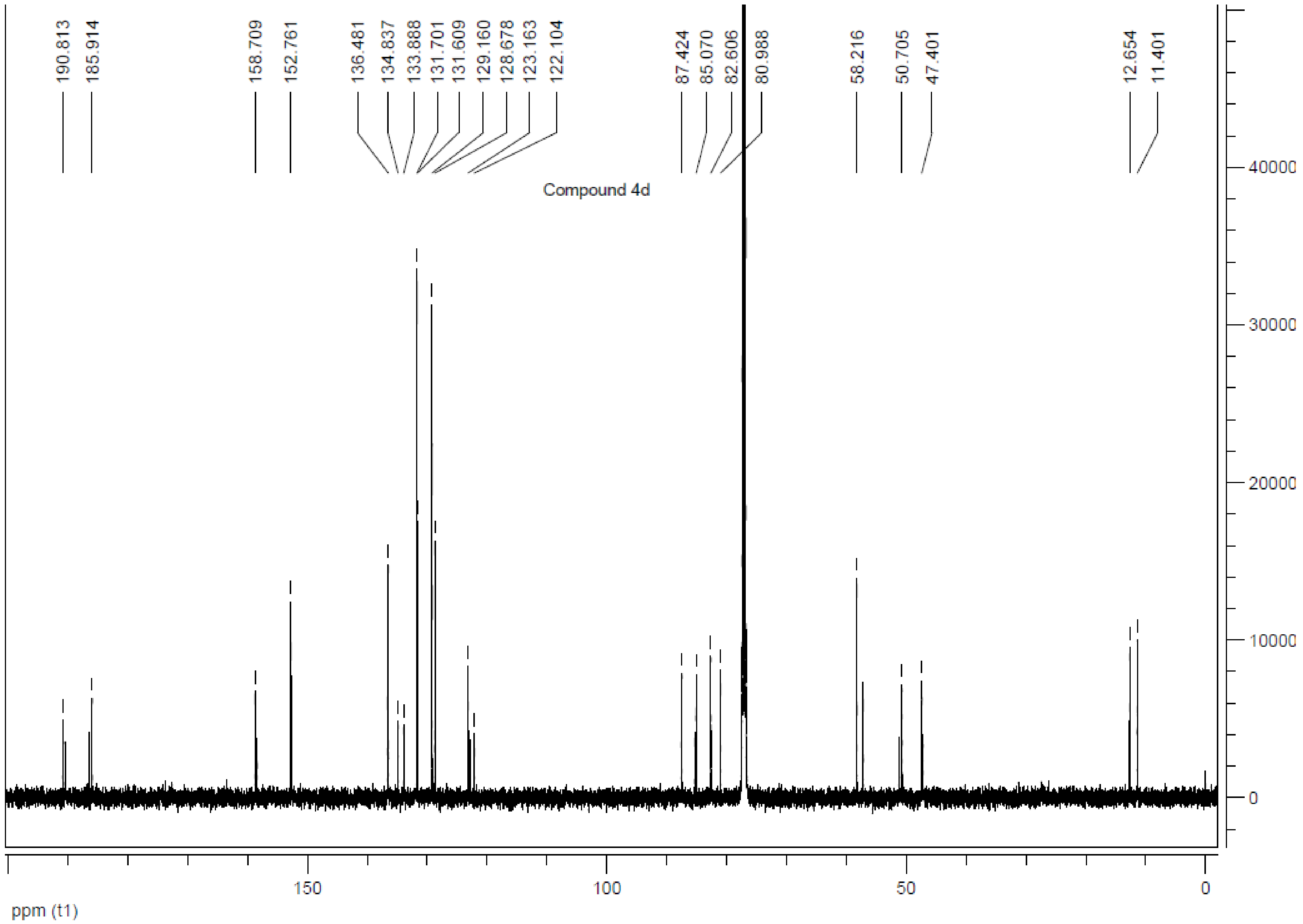
190.813
185.914
158.709
152.761
136.481
134.837
133.888
131.701
131.609
129.160
128.678
123.163
122.104
87.424
85.070
82.606
80.988
58.216
50.705
47.401
12.654
11.401
Compound 4d
40000
30000
20000
10000
0
150
100
50
0
ppm (t1)

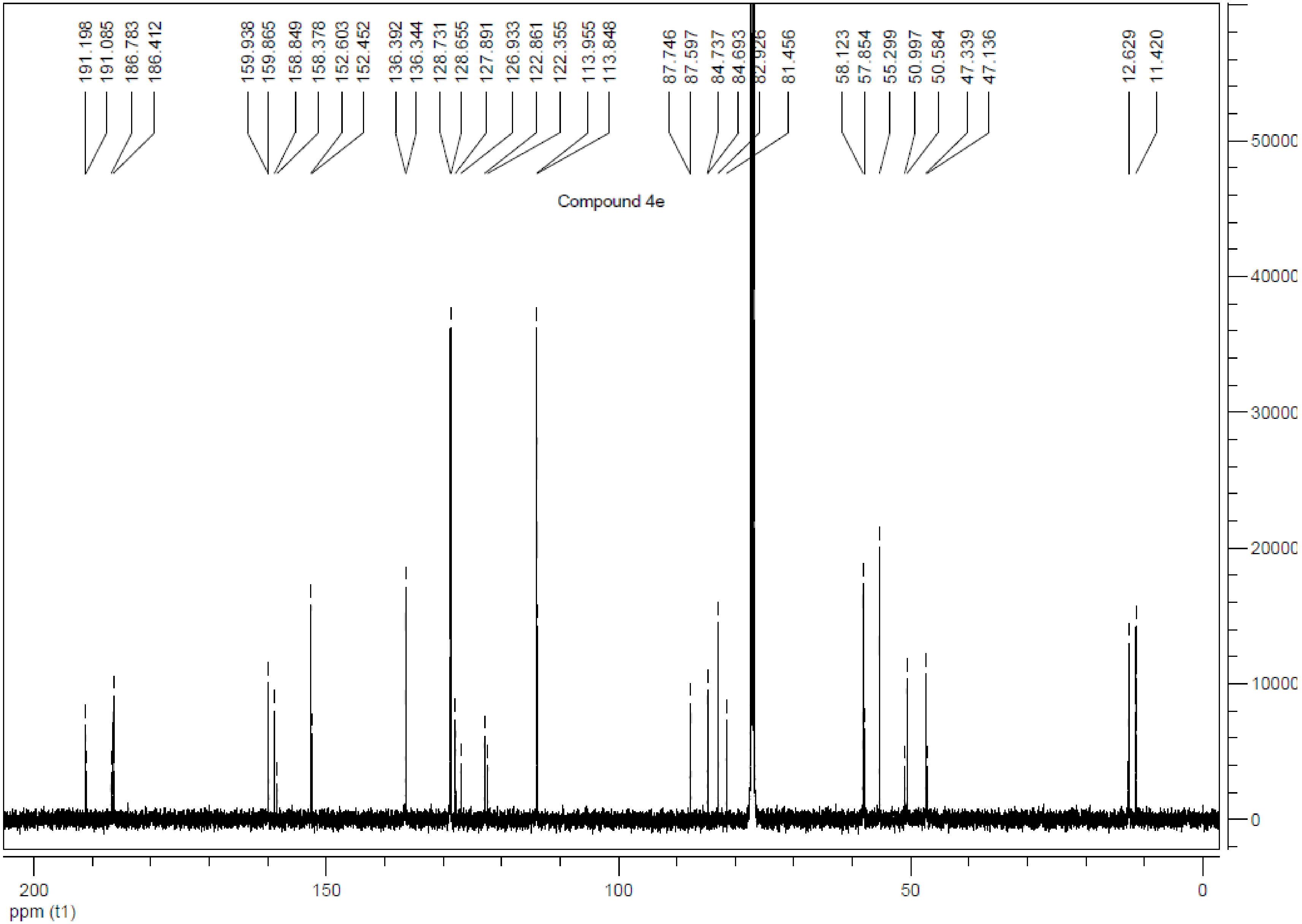
191.198
191.085
186.783
186.412
159.938
159.865
158.849
158.378
152.603
152.452
136.392
136.344
128.731
128.655
127.891
126.933
122.861
122.355
113.955
113.848
87.746
87.597
84.737
84.693
82.925
81.456
58.123
57.854
55.299
50.997
50.584
47.339
47.136
12.629
11.420
Compound 4e
50000
40000
30000
20000
10000
0
200
150
100
50
0
ppm (t1)

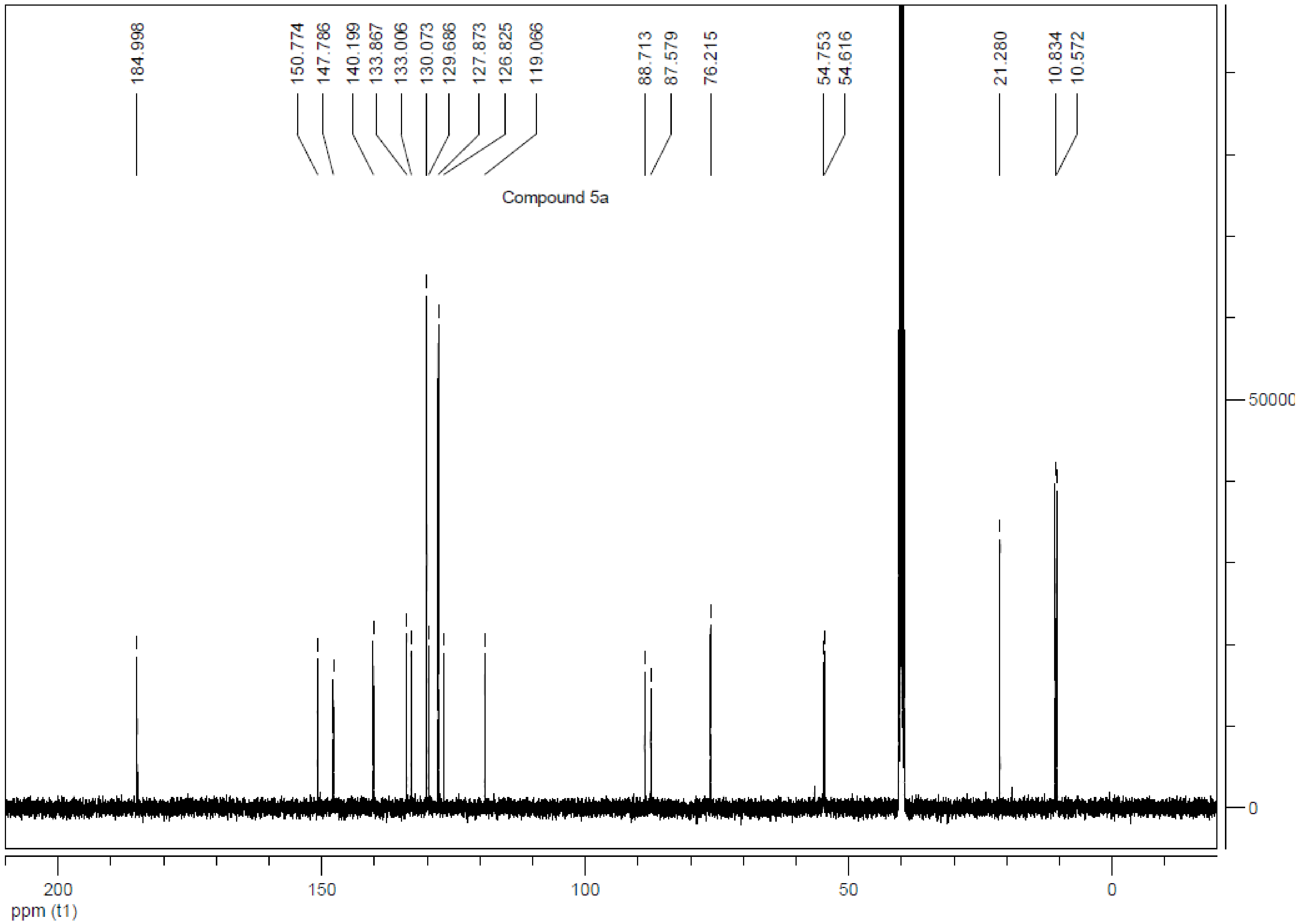
184.998
150.774
147.786
140.199
133.867
133.006
130.073
129.686
127.873
126.825
119.066
88.713
87.579
76.215
54.753
54.616
21.280
10.834
10.572
Compound 5a
50000
0
200
150
100
50
0
ppm (t1)

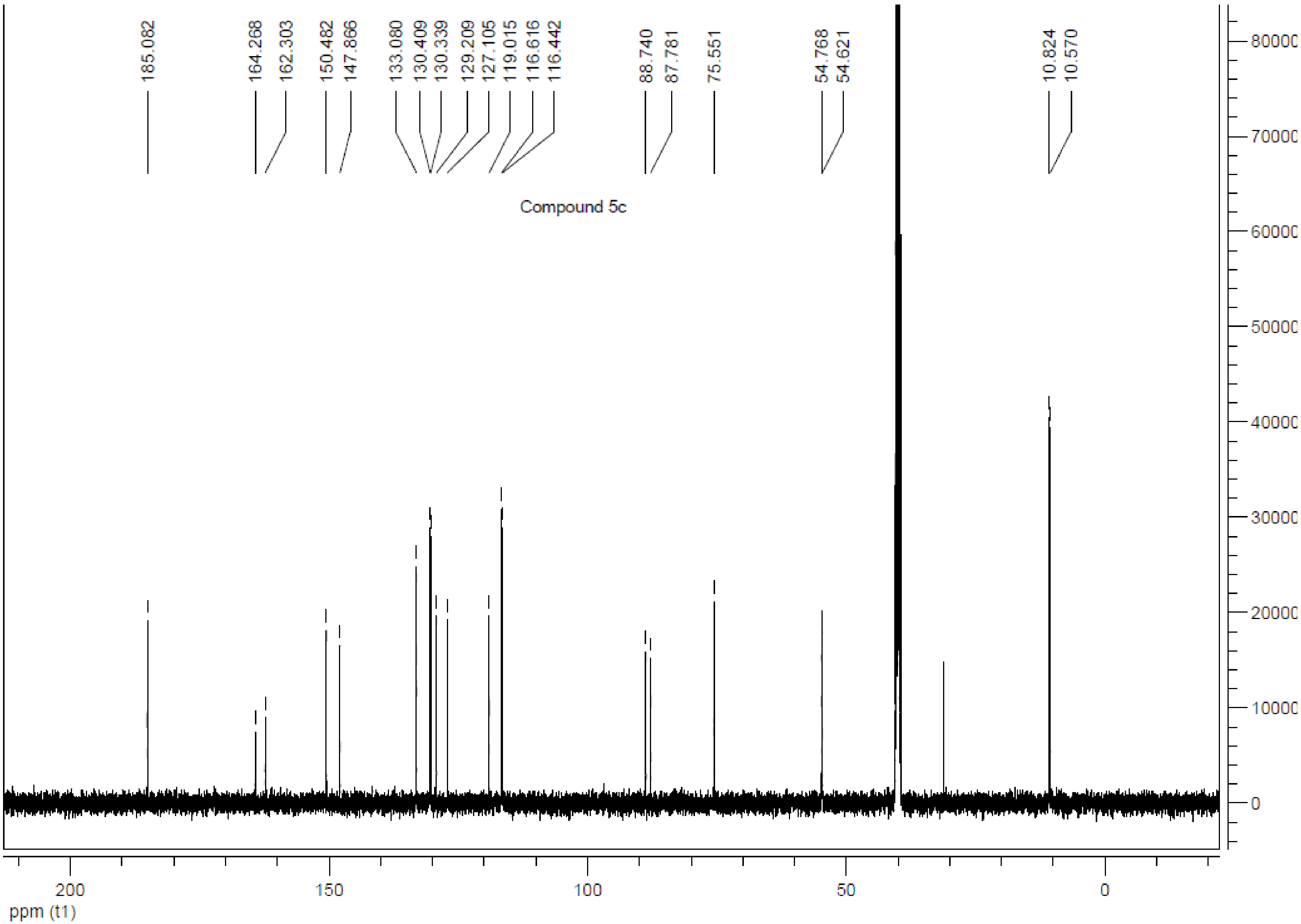
185.082
164.268
162.303
150.482
147.866
133.080
130.409
130.339
129.209
127.105
119.015
116.616
116.442
88.740
87.781
75.551
54.768
54.621
10.824
10.570
Compound 5c
80000
70000
60000
50000
40000
30000
20000
10000
0
200
150
100
50
0
ppm (t1)

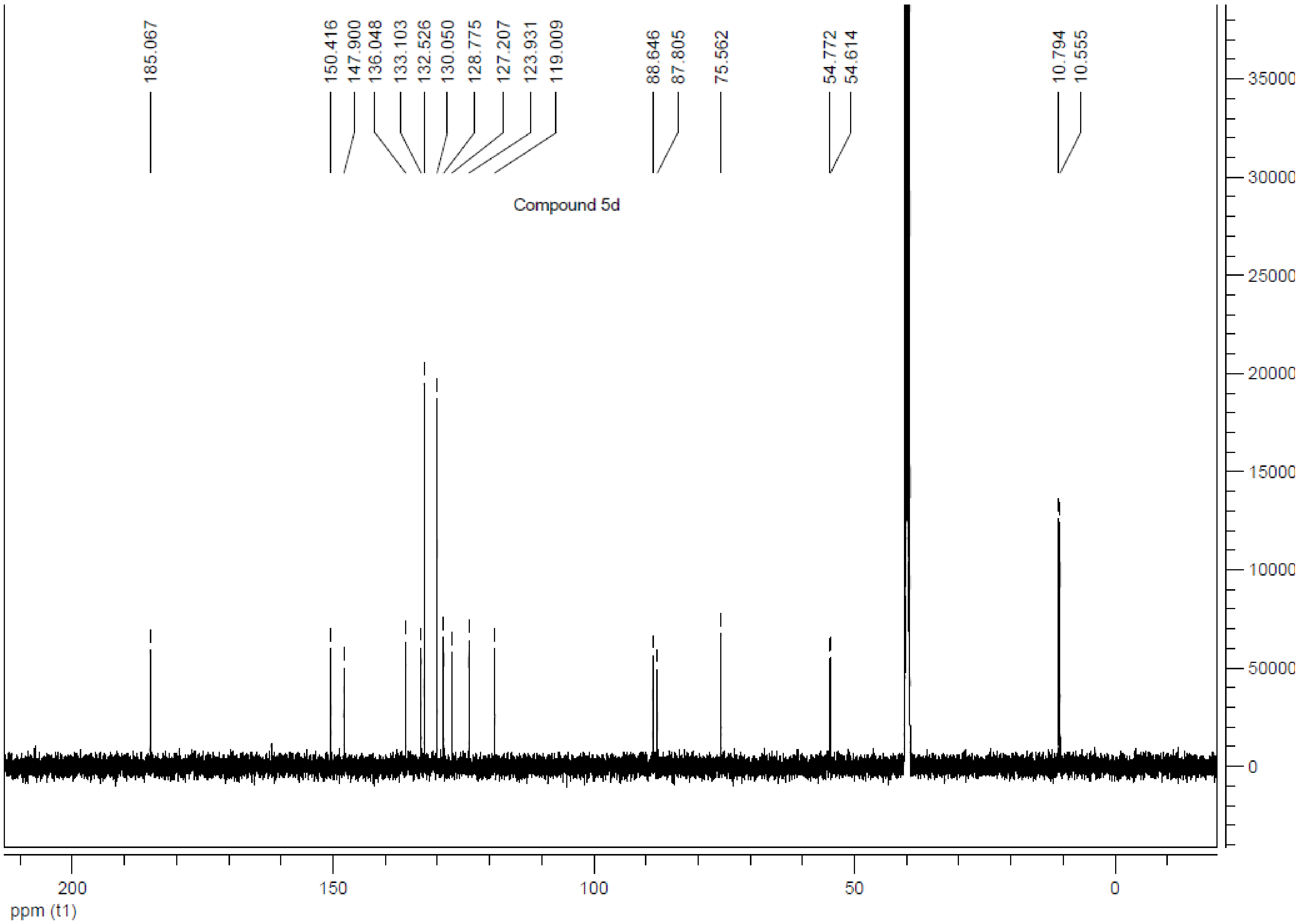
185.067
150.416
147.900
136.048
133.103
132.526
130.050
128.775
127.207
123.931
119.009
88.646
87.805
75.562
54.772
54.614
10.794
10.555
Compound 5d
35000
30000
25000
20000
15000
10000
50000
0
200
150
100
50
0
ppm (t1)

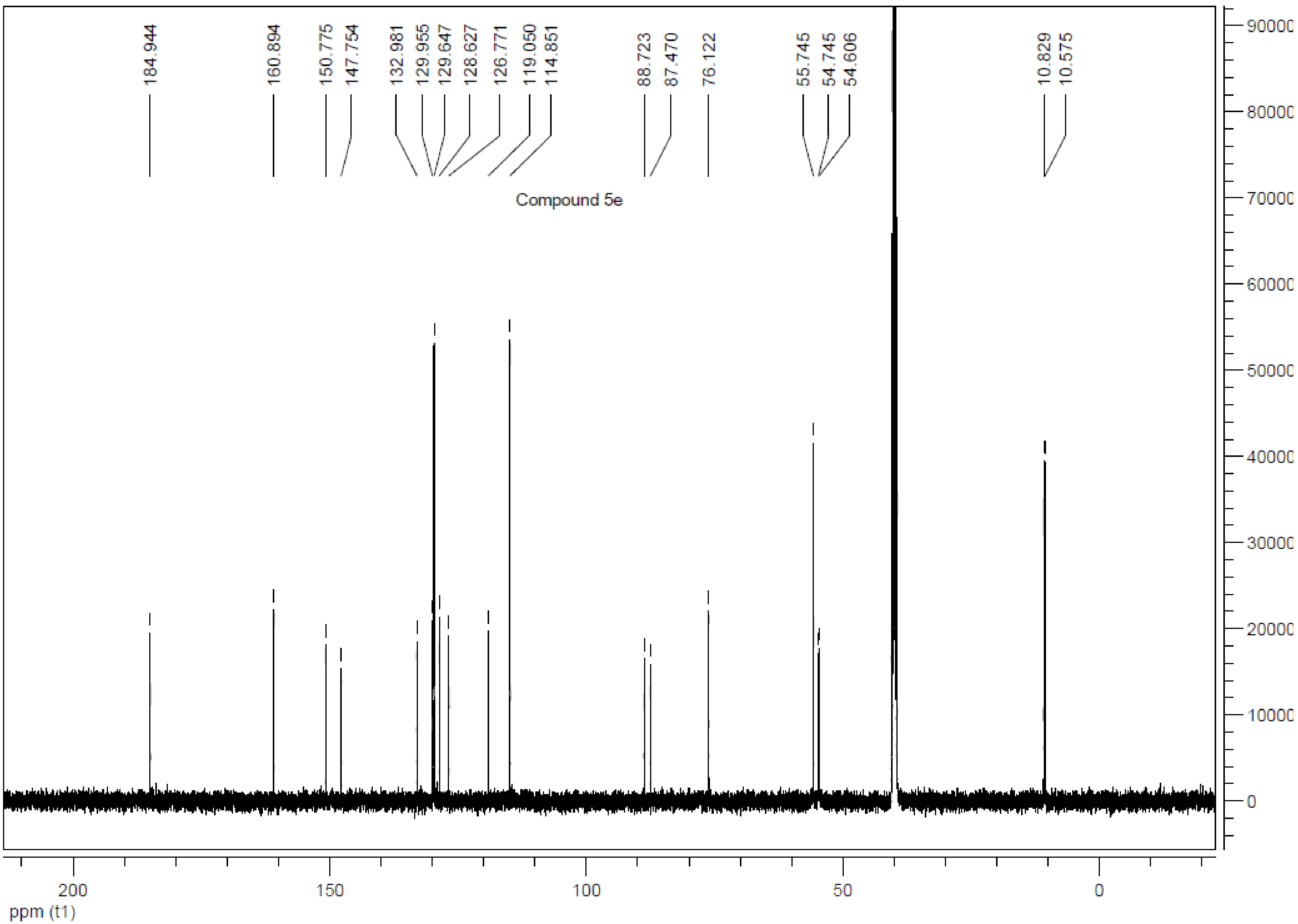
184.944
160.894
150.775
147.754
132.981
129.955
129.647
128.627
126.771
119.050
114.851
88.723
87.470
76.122
55.745
54.745
54.606
10.829
10.575
Compound 5e
ppm (t1)
